# Relative strengths in daily living skills among autistic individuals and individuals with related developmental conditions who have co‐occurring intellectual disability

**DOI:** 10.1111/jcpp.70124

**Published:** 2026-01-28

**Authors:** Elaine B. Clarke, Catherine Lord, Vanessa Hus Bal

**Affiliations:** ^1^ Graduate School of Applied and Professional Psychology Rutgers, The State University of New Jersey Piscataway NJ USA; ^2^ Department of Psychiatry and Biobehavioral Sciences University of California, Los Angeles Los Angeles CA USA

**Keywords:** Daily living skills, autism spectrum disorder, intellectual disability, mental health, longitudinal research

## Abstract

**Background:**

Strong daily living skills (DLS) are associated with positive outcomes. Prior studies have documented intellectual quotient (IQ)‐DLS discrepancies in autistic individuals with average or higher cognitive abilities. Little work in this area includes individuals with co‐occurring intellectual disability (ID) or examines IQ‐DLS discrepancies at the level of DLS subdomains (i.e., Personal, Domestic, and Community skills). This study examined trajectories of IQ‐DLS discrepancies from ages 2–25 in autistic individuals with ID.

**Methods:**

A total of 127 individuals from a well‐characterized longitudinal cohort with verbal IQ < 70 at age 9 were included. IQ‐DLS discrepancy scores were calculated by subtracting DLS AEs from nonverbal mental age (NVMA) estimates. Group‐based trajectory modeling identified IQ‐DLS discrepancy trajectory groups for the DLS domain and Personal, Domestic, and Community subdomains. One‐way ANOVA and chi‐square analyses were used to compare trajectory groups on demographic and phenotypic characteristics.

**Results:**

Two DLS domain discrepancy trajectory groups emerged: IQ > DLS (cognitive abilities exceeded DLS) and IQ < DLS (DLS exceeded cognitive abilities); most participants (78%) were in the IQ > DLS group. An additional group, IQ = DLS (cognitive abilities and DLS were commensurate), emerged in each of the DLS subdomains, for a total of three trajectory groups. Within DLS subdomains, approximately 80% of participants were in either the IQ = DLS or the IQ < DLS trajectory group. In other words, examining scores at the DLS domain‐level indicated most participants had cognitive abilities that exceeded DLS, but subdomain scores indicated most participants had DLS that equaled or exceeded cognitive abilities.

**Conclusions:**

These results challenge the notion that autism is usually associated with weaknesses in DLS compared to IQ. At the subdomain level, 80% of participants had DLS commensurate with or stronger than their cognitive abilities, indicating domain‐level scores may obscure important variability in daily functioning. This work highlights the importance of including autistic individuals with ID in research; patterns observed in samples without ID may not be generalizable.

## Introduction

Independence in life skills can support positive outcomes. This is well‐documented in the general population (Hermens, Super, Verkooijen, & Koelen, 2017; Schoon, Nasim, Sehmi, & Cook, 2015) and in clinical populations, including autism spectrum disorder (ASD; referred to throughout as “autism”) and related conditions (Clarke, McCauley, & Lord, 2021; Laxman, Taylor, DaWalt, Greenberg, & Mailick, 2019).

Prior research suggests autistic individuals exhibit lower daily living skills (DLS) than expected based on estimated cognitive ability (i.e., intellectual quotient [IQ]; Kanne et al., 2011). IQ‐DLS discrepancies are well‐documented in autistic individuals with average or higher IQ (Duncan & Bishop, 2015; Klin et al., 2007). Multiple studies report discrepancies of one standard deviation or more between standardized IQ and DLS scores (Duncan & Bishop, 2015; Kraper, Kenworthy, Popal, Martin, & Wallace, 2017; McQuaid et al., 2021). Discrepancies appear to be moderately associated with higher internalizing and externalizing symptoms and weaker executive function, though not consistently related to demographic variables such as age, sex, or caregiver education (Braverman, Edmunds, Hastedt, & Faja, 2024; Donoso et al., 2024; Gardiner, 2018).

Few studies in this area include autistic individuals with a range of cognitive abilities (see Alvares et al., 2019, Kanne et al., 2011 for exceptions). U.S. prevalence estimates indicate almost 40% of autistic individuals have co‐occurring intellectual disability (ID; Shaw, 2025), yet this subgroup remains underrepresented in research (Russell et al., 2019). Autistic individuals with ID may also have IQ‐DLS discrepancies, but the magnitude and direction of discrepancies could differ due to distinct developmental trajectories of cognitive and adaptive functioning. One cross‐sectional study found that autistic adults with an IQ < 85 had DLS approximately one standard deviation lower than their cognitive abilities; in contrast, adults with an IQ > 85 had DLS approximately two standard deviations below their cognitive abilities (Teh, Vo, & Bal, 2024).

The Vineland Adaptive Behavior Scales (VABS‐3; Sparrow, Cicchetti, & Saulnier, 2016) is a widely used measure of DLS. The VABS produces age‐standardized adaptive behavior composite (ABC) and DLS domain scores, and standardized scores and age equivalents (AEs) for three DLS subdomains: personal (e.g., toileting, bathing, and grooming), domestic (e.g., cooking, household chores), and community (e.g., obeying traffic signals, using money, ordering in a restaurant); see Glover, Liddle, Fassler, and Duncan (2023) for detailed descriptions of DLS subdomains. Existing studies have not examined IQ‐DLS discrepancies by DLS subdomains. It is unclear whether IQ‐DLS discrepancies vary across Personal, Domestic, and Community skills or whether IQ‐DLS discrepancies reported in the literature are driven by one subdomain of skills.

Existing studies of IQ‐DLS discrepancies in autism are cross‐sectional, providing little insight into how IQ‐DLS discrepancies may change or remain stable over time (Alvares et al., 2019; Duncan & Bishop, 2015; Kanne et al., 2011). These studies indicate IQ‐DLS discrepancies are apparent from late childhood through adulthood, but it remains unclear whether these patterns reflect developmental change or stable individual differences (Alvares et al., 2019; McQuaid et al., 2021). Longitudinal examinations of DLS only suggest VABS DLS raw scores and AEs increase across childhood and adolescence (though more slowly than in the general population) and plateau in adulthood (Baghdadli et al., 2012, 2018; Freeman, Del' PHomme, Guthrie, & Zhang, 1999). High IQ and lower scores on measures of autistic features in childhood are positively associated with DLS growth (Bal, Kim, Cheong, & Lord, 2015; Szatmari et al., 2015). Longitudinal studies of cognitive ability only indicate IQ increases with age (Anderson, Liang, & Lord, 2014); stronger language abilities and fewer autistic features in childhood are associated with IQ growth (Simonoff et al., 2020). However, no studies have examined how IQ and DLS change relative to one another over time, particularly in autistic individuals with ID.

### The current study

This study examines trajectories of IQ‐DLS discrepancies from 2 to 25 years in autistic individuals with co‐occurring ID. Discrepancies are examined separately by DLS subdomains (i.e., Personal, Domestic, Community) to identify patterns that may be obscured by examining composite domain‐level scores alone. This work builds upon prior longitudinal studies that have separately considered growth in DLS and growth in IQ by examining how IQ and DLS vary from one another over time.

*Aim 1. Calculate trajectories of IQ‐DLS discrepancy scores for the VABS DLS domain and subdomains (*i.e., *Personal, Domestic, Community) from ages 2–25 in a sample of autistic individuals with co‐occurring ID*. Based on prior cross‐sectional research (Alvares et al., 2019; Teh et al., 2024), it is hypothesized that IQ‐DLS discrepancy scores will increase (i.e., IQ will be stronger than DLS) from early childhood through adolescence and stabilize or decrease (i.e., IQ and DLS will be comparable or DLS will be stronger than IQ) in adulthood.

*Aim 2. Describe the phenotypic characteristics associated with inclusion in the identified IQ‐DLS discrepancy groups*. It is hypothesized that higher IQ and fewer autism features will be associated with inclusion in IQ‐DLS discrepancy trajectory groups with higher discrepancy scores (i.e., stronger IQ than DLS). In line with prior work in autistic individuals with average or higher cognitive abilities, it is expected that inclusion in trajectory groups with higher discrepancy scores (i.e., stronger IQ than DLS) will be associated with more internalizing and externalizing symptoms.

## Method

### Participants

Consecutive referrals (*N* = 213) under 37 months old to clinics in North Carolina and Chicago were enrolled in a longitudinal cohort.^1^ At the age of 9, 40 children from Michigan with similar age and diagnostic characteristics joined the cohort, resulting in a total of 253 participants. Detailed descriptions of this sample can be found in prior work (Lord et al., 2006). These analyses included 127 participants with (a) a verbal IQ score of less than 70 at age 9 and (b) data on IQ and DLS from at least two time points between ages 2 and 25 years (*M* time points = 3.94, *SD* = 1.16); see Appendix S1 for comparisons between the longitudinal cohort and this sample.

Seventeen participants in the current study have never been diagnosed with autism, despite repeated assessments. Those with and without ASD diagnoses in this cohort share many similarities across development (Lord, McCauley, Pepa, Huerta, & Pickles, 2020). Thus, participants who never received an ASD diagnosis were retained in the current analyses. See Appendix S2 for analyses excluding participants who never received an ASD diagnosis (Table S1, Figure S1).

### Procedures

Questionnaires and direct testing, including the Autism Diagnostic Observation Schedule (ADOS; Lord, Risi, Lambrecht, Goode, & McInnes, 2000; Lord et al., 2012), IQ tests chosen from a standard hierarchy (Anderson et al., 2014), and parent interviews conducted by trained clinicians, including the VABS (Sparrow, Balla, & Cicchetti, 1984; Sparrow, Cicchetti, & Balla, 2005), were completed at approximately ages 2, 3, 5, 9, 18, and 25. Ethical approval was obtained from the Institutional Review Boards at UCLA, University of Michigan, University of Chicago, and University of North Carolina, Chapel Hill, as appropriate. Parents and participants over 18 years who were their own legal guardians gave written consent as required by the relevant institutional review board(s) prior to visits.

### Measures

#### Autism features

The ADOS (Lord et al., 2000, 2012) is a semistructured, clinician‐administered assessment measuring social communication impairments and restricted and repetitive behaviors (RRBs). The ADOS produces a total calibrated severity score (CSS; Gotham, Pickles, & Lord, 2009) and CSS for social affect (SA) and restricted and RRB (Hus, Gotham, & Lord, 2014). CSS ranges from 1 to 10; higher scores indicate more difficulties in social communication and/or clearer RRB during the ADOS. ADOS scores from age 9 (*M*_age_ = 9.03, *SD* = 2.23, range = 3.67–11.92) are used to describe the sample (Table 1), as prior work indicates autism features are relatively stable after mid‐childhood (Elias & Lord, 2022).

**Table 1:** Descriptive characteristics of the current sample

	*n* = 127
Demographic characteristics	
Sex (% female)	23.6
Diagnostic status (% DD^a^)	11.8
Race	
% White	63.8
% African American/Black	33.9
% Asian/Pacific Islander	1.6
% American Indian	0.7
% Multiracial	0.9
Urbanicity (% urban)	48.8
Caregiver Education (% college education)	47.2
Language level (% Nonspeaking or minimally speaking)	84.7
Phenotypic characteristics at age 9	
Verbal IQ	29.24 (17.42)
Nonverbal IQ	43.39 (21.85)
Vineland ABC^b^	34.68 (11.60)
ADOS CSS^c^	7.61 (2.04)
ADOS CSS – SA^d^	7.50 (1.94)
ADOS CSS – RRB^e^	7.73 (1.89)
Profound categorization (% profound autism)	77.9

At age 9, ratio IQ scores were calculated for 24.4% of participants (nonverbal IQ) and 27.5% of participants (verbal IQ) who completed IQ tests outside of age norms. Standardized IQ scores from age 9 are reported for all other participants, and nonverbal mental age was used to calculate all discrepancy trajectories. IQ, intelligent quotient.

^a^ Nonspectrum developmental disability.

^b^ Adaptive behavior composite standard scores.

^c^ Autism Diagnostic Observation Schedule, calibrated severity score.

^d^ Social affect.

^e^ Restricted, repetitive behaviors.

#### Language level

Language level was derived from ADOS item A1, Overall Level of Non‐Echoed Language from participants' most recent ADOS (*M*_age_ = 16.91, *SD* = 4.91 years). Information from A1 codes was collapsed into a binary variable: 1 = complex speech (defined as utterances with two or more clauses), 0 = non‐or‐minimally speaking (defined as use of two‐ or three‐word phrases or single words without complex speech).

#### Cognitive ability

The Mullen Scales of Early Learning (MSEL; Mullen, 1995) were administered at age 2. Later cognitive assessments were chosen from a standard hierarchy, including the Wechsler Intelligence Scale for Children (WISC‐III; Wechsler, 1991), Wechsler Abbreviated Scale of Intelligence (WASI; Wechsler, 1999; 2011), Differential Abilities Scale (DAS; Elliott, 1990; 2007), and MSEL. AEs from nonverbal (NV) domain subtests were averaged to calculate NV abilities. To provide context for the assessment methods used to estimate NV mental age, Table 2 summarizes the most advanced cognitive test administered within the hierarchy for each participant.

**Table 2:** Most advanced cognitive test in hierarchy administered by Daily living skills (DLS) subdomain trajectory groups

Cognitive test	DLS		Personal			Domestic			Community			Total
	IQ<DLS	IQ>DLS	IQ<DLS	IQ = DLS	IQ>DLS	IQ<DLS	IQ = DLS	IQ>DLS	IQ<DLS	IQ = DLS	IQ>DLS	
	*n* = 24	*n* = 100	*n* = 16	*n* = 97	*n* = 16	*n* = 17	*n* = 94	*n* = 18	*n* = 10	*n* = 95	*n* = 24	
% MSEL^1^	2.0	52.0	**25.0** ^**a**^	**53.2** ^**b**^	**0** ^**a**^	17.6	47.2	44.4	**0** ^**a**^	**55.4** ^**b**^	**12.5** ^**a**^	54
% DAS^2^ – Early Years	12.0	25.0	68.8	28.7	56.3	29.4	38.4	38.9	80	30.4	45.8	47
% DAS^2^ – School Age	4.0	4.0	6.2	4.3	18.7	17.6	2.2	16.7	10	4.3	12.5	8
% WASI^3^	3.0	2.0	0	3.2	12.5	23.6	1.1	0	0	1.1	16.7	5
% Other^4^	3.0	7.0	0	8.5	12.5	11.8	8.8	0	10	6.5	12.5	10

All participants were administered the MSEL at age 2. At subsequent visits, participants were administered the most advanced cognitive assessment appropriate for their current ability level; tests were selected from a standard hierarchy (Anderson et al., 2014). The most advanced cognitive test ever administered to each participant is depicted here. Text in bold indicates significant group differences after corrections for multiple comparisons. ^a,b^For each trajectory group, mean values without common superscripts are significantly different (*p* < .05).

^1^ Mullen Scales of Early Learning (Mullen, 1995).

^2^ Differential Ability Scales (Elliot, 1990; 2007).

^3^ Wechsler Abbreviated Scale of Intelligence (Wechsler, 1991; 2011).

^4^ Other administered IQ cognitive tests included the Merrill‐Palmer‐Revised Scales of Development (M‐P‐R; Roid & Sampers, 2004; *n* = 5), the Raven Progressive Matrices (Raven & Raven, 2003; *n* = 3), and the Wechlser Intelligence Scale for Children (WISC‐III; Wechlser, 1991; *n* = 1).

Verbal and NA standardized IQ scores from age 9 are used to describe the sample (Table 1); prior work suggests IQ remains relatively stable after mid‐childhood (Pickles, McCauley, Pepa, Huerta, & Lord, 2020). Ratio IQs (Bishop, Farmer, & Thurm, 2015) were calculated when tests were administered outside the standardized age range. The proportion of participants meeting criteria for profound autism (i.e., IQ < 50, limited or no functional speech, and substantial support needs in daily functioning) is also reported in Table 1. These individuals were also included in the analyses reported in the Lancet Commission, which first proposed the terminology of profound autism (Lord et al., 2022).

#### Daily living skills

The Vineland Adaptive Behavior Scales (VABS; Sparrow et al., 1984, 2005) comprehensive clinician‐interview form, was used to assess adaptive functioning. Participants completed the first edition (Sparrow et al., 1984) from ages 2–9 and the second edition (Sparrow et al., 2005) from ages 14–25. The VABS assesses adaptive behaviors in three primary domains: Communication, DLS, and Socialization. The VABS produces domain‐specific standard scores, as well as an overall ABC standard score. VABS ABC standard scores from age 9 are used to describe the current sample (Table 1).

The DLS domain of the VABS comprises three subdomains: Personal, Domestic, and Community. Each subdomain produces an age equivalent (AE) estimate. The Personal subdomain assesses DLS related to self‐care, including dressing, bathing, and physical health. The Domestic subdomain assesses DLS related to household maintenance, including chores, meal preparation, and laundry. The Community subdomain assesses DLS related to navigating public spaces, including making purchases, running errands, and using public transportation. AEs from each of the DLS subdomains were used to calculate separate IQ‐DLS discrepancy scores for Personal, Domestic, and Community skills.

#### IQ‐DLS discrepancy scores

IQ‐DLS discrepancies were calculated by subtracting VABS DLS subdomain AEs from non‐verbal mental age, herein referred to as NV abilities. Because age‐standardized IQs and/or cognitive estimates are not available for most participants, particularly at later ages, AEs from the VABS and cognitive tests were used to calculate discrepancies. Although ratio IQs can be computed using cognitive test AEs divided by chronological age, these tend to decrease as participants get older (Bishop et al., 2015). Thus, the use of AEs and NV abilities avoids artificial deflation of ratio IQs, which would directly impact the discrepancy calculation.

Four IQ‐DLS discrepancies were calculated for each timepoint, one for the DLS domain, and one each for the Personal, Domestic, and Community subdomains. Participants needed to have both IQ and DLS data to calculate an IQ‐DLS discrepancy score for a given time point. Positive discrepancy scores indicate that DLS data are lower than expected based on NV abilities (IQ > DLS). Negative scores indicate that DLS data are higher than expected based on NV abilities (IQ < DLS), and scores close to zero indicate comparable DLS and NV abilities (IQ = DLS).

### Data analysis

Group‐based trajectory modeling was performed using the traj plugin in Stata 16 (Jones & Nagin, 2007, 2013). Trajectory models were estimated using all available data under a missing‐at‐random (MAR) assumption, consistent with standard maximum‐likelihood estimation in group‐based trajectory modeling (Nagin, 2005). This approach allows participants with incomplete data to contribute to parameter estimation without requiring imputation. Given increasing missing data at later assessments, we report the proportion of missing data at each time point (Table 3). The best‐fitting model and number of trajectory groups were determined using Bayesian Information Criteria (BIC). Unconditional 1, 2, 3, 4, and 5 class models were compared using Bayesian Information Criterion (BIC) and the smallest group membership percentage (Tables S1 and S2). After classes were determined, higher‐order effects were tested to establish whether cubic, quadratic, linear, or intercept modeling best explained variation over time. Entropy values and posterior probabilities were also used to assess adequate model fit (Nagin, Jones, Passos, & Tremblay, 2018).

**Table 3:** Average IQ‐DLS discrepancy score by timepoint and DLS domain and subdomain trajectory groups

	Data collection time point					
	**2**	**3**	**5**	**9**	**18**	**25**
*n*	115	102	77	98	73	40
Proportion of missing data	7.3%	17.7%	37.9%	21.0%	41.1%	67.7%
	**IQ‐DLS discrepancy score** ***M* (*SD*) [range]**					
	**DLS domain**					
IQ > DLS	4.68 (4.53) [0.19–10.0]	6.36 (4.53) [−3.6–16.0]	16.84 (13.49) [−2.80–45.54]	28.73 (17.01) [−3.0–63.10]	35.33 (21.15) [4.0–80.0]	23.33 (16.32) [7.0–55.91]
IQ = DLS	1.09 (4.24) [−15.41–13.50]	3.34 (4.57) [−5.84–14.60]	4.19 (7.85) [−14.62–26.0]	3.66 (11.97) [−18.0–46.0]	−24.61 (26.49) [−102.0–12.0]	−2.66 (14.30) [−31.67–46.0]
	**Personal subdomain**					
IQ > DLS	4.19 (6.77) [−15.41–10.16]	6.86 (5.26) [−4.6–16.0]	18.59 (15.67) [−3.79–41.54]	37.36 (17.02) [5.0–64.04]	51.72 (25.93) [13.0–97.26]	18.50 (35.31) [−13.0–61.0]
IQ = DLS	1.58 (4.07) [−7.61–14.50]	2.40 (4.29) [−6.62–13.36]	3.81 (7.71) [−10.28–23.0]	3.88 (12.61) [−29.80–42.0]	−3.24 (15.67) [−60–24.0]	−7.16 (10.45) [−23.0–12.0]
IQ < DLS	1.31 (3.45) [−5.79–9.36]	0.21 (4.38) [−8.84–5.19]	1.37 (8.27) [−12.62–15.92]	4.62 (15.92) [−23.0–34.0]	−37.81 (27.35) [−84.0–11.0]	−51.25 (13.88) [−77.0 to −32.0]
	**Domestic subdomain**					
IQ > DLS	3.04 (4.27) [−6.20–9.0]	5.69 (5.47) [−3.20–18.0]	14.79 (11.16) [0.20–36.0]	32.38 (15.88) [4.0–58.10]	39.31 (33.45) [−4.20–110.26]	33.40 (32.81) [5.0–63.0]
IQ = DLS	−0.07 (5.17) [−10.68–12.35]	3.37 (5.37) [−8.30–15.36]	5.83 (11.62) [−17.62–49.54]	5.05 (16.06) [−35.0–64.0]	−18.16 (21.55) [−55.0–38.0]	−19.43 (21.32) [−71.0–16.0]
IQ < DLS	−1.99 (7.18) [−19.41–8.65]	0.20 (11.24) [−33.40–10.50]	2.82 (4.70) [−5.54–10.80]	−2.14 (15.12) [−27.0–29.08]	−77.76 (33.70) [−145.0 to −31.0]	−72.38 (22.65) [−102.0 to −41.0]
	**Community subdomain**					
IQ < DLS	11.07 (4.21) [1.56–17.36]	11.55 (5.16) [3.09–19.80]	25.35 (16.83) [2.20–60.54]	37.75 (16.09) [13.0–67.68]	37.73 (16.09) [13.0–67.68]	27.43 (39.77) [−20.0–97.6]
IQ = DLS	5.81 (5.65) [−9.41–18.52]	7.96 (5.54) [−9.86–20.20]	9.83 (9.06) [−5.6–42.0]	10.76 (12.82) [−20.35–53.08]	−6.54 (12.82) [−33.0–26.0]	−10.88 (12.04) [−34.0–19.0]
IQ > DLS	5.97 (6.83) [−0.68–23.13]	5.87 (6.95) [−4.50–15.90]	8.74 (12.49) [−15.62–19.46]	4.35 (9.20) [−11.6–20.25]	−67.80 (31.89) [−134.0 to −41.0]	−41.33 (11.71) [−57.0 to −27.0]

Positive IQ‐DLS discrepancy scores indicate NV abilities are higher than the age equivalence score for the given DLS subdomain. Negative IQ‐DLS scores indicate the age equivalence score for the given DLS subdomain is higher than the nonverbal mental age. An IQ‐DLS discrepancy score of zero or close to zero indicates that NV abilities and DLS AE scores are equal. Tables S3–S5 present the mean, standard deviation, and range of NV abilities and DLS AEs by DLS subdomain trajectory groups. AE, age equivalent; IQ‐DLS, intellectual quotient‐daily living skills; NV, nonverbal.

One‐way ANOVA and chi‐square analyses were used to compare trajectory groups on demographic and phenotypic characteristics (e.g., sex, race, caregiver education, language level, and ADOS CSS, IQ, and VABS ABC scores from age 9). Associations between trajectory group membership and internalizing and externalizing were also examined; see Appendix S3. The Benjamini‐Hochberg procedure was used to correct for multiple comparisons.

## Results

### Aim 1: IQ‐DLS discrepancy trajectory groups

**Figure 1:**
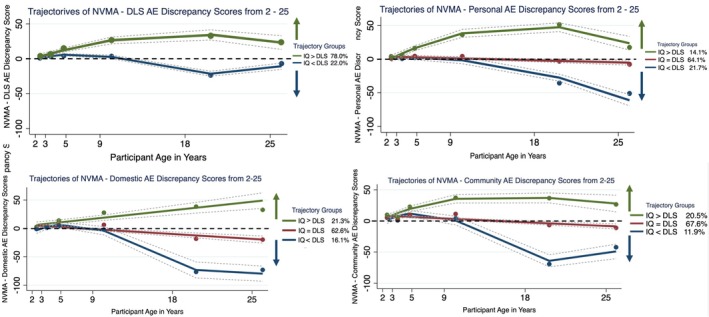
Trajectories of NV abilities—DLS AE domain and subdomain discrepancy scores from ages 2–25. DLS AE, daily living skills age equivalent; NV, nonverbal

For the DLS domain, a two‐group model best fit the data (Table S2). More than three‐quarters of participants (78%) had IQ‐DLS discrepancy scores near‐zero from ages 2–9, indicating childhood growth in NV abilities and DLS was commensurate (referred to below as IQ = DLS). Discrepancy scores slightly decreased from ages 9–18 and remained stable from ages 18–25, suggesting DLS growth marginally outpaced NV abilities growth in adolescence and adulthood. IQ‐DLS discrepancy scores in the remaining 22% of the sample increased with age, indicating growth in NV abilities outpaced growth in DLS at all ages (referred to below as IQ > DLS; Figure 1).

In contrast, for all three subdomains (Personal, Domestic, Community), a three‐group model best fit the data (Table S2). Approximately two‐thirds of participants had commensurate NVMA and DLS AEs across time, as indicated by IQ‐DLS discrepancy scores of zero or near‐zero (IQ = DLS). Within each DLS subdomain, two other trajectory groups emerged. The first group had negative discrepancies that tended to get more negative over time, indicating NVMA was lower than DLS AEs (referred to below as IQ < DLS groups). The second group had positive discrepancies that tended to get more positive, indicating NVMA was higher than DLS AEs (IQ > DLS). These groups comprised anywhere from 10% to 20% of the sample for each subdomain (Figure 1). Within‐group discrepancy magnitudes ranged widely (e.g., median absolute differences = 18–54 months across subdomains; see Table 3), suggesting variability in the degree to which cognitive and adaptive skills diverged (see also Tables S3–S5). Participants' individual growth patterns across the three subdomains tended to be similar; 58% of participants were in the same trajectory group across all three DLS subdomains, 34% were in the same trajectory group for two subdomains, and 8% were in different trajectory groups for each subdomain. In other words, for most participants, the overall pattern of IQ‐DLS discrepancies was stable across domains. Entropy values for all models ranged from .67–.84, indicating moderate classification certainty (Table S2).

Growth patterns for the Personal and Community subdomains were comparable when only autistic participants were included in the analyses (Figure S1). For the Domestic subdomain, the data from autistic participants only was best described by a two‐group model, an IQ = DLS group and an IQ < DLS group (there was no IQ > DLS group; Table S1).

#### Personal subdomain

The IQ < DLS trajectory group comprised 14.1% of the sample, and the slope of the IQ < DLS trajectory was quadratic. The IQ = DLS group comprised 61.1% of the sample, and the slope of the IQ = DLS group was linear. Finally, the IQ > DLS group comprised 21.7% of the sample, and the slope of the IQ > DLS group was quadratic (Figure 1).

#### Domestic subdomain

The IQ < DLS trajectory group comprised 16.1% of the sample, and the slope of the trajectory was cubic. The IQ = DLS group comprised 62.6% of the sample, and the slope of the IQ = DLS group was linear. Finally, the IQ > DLS group comprised 21.3% of the sample, with a linear slope.

#### Community subdomain

The IQ < DLS trajectory group comprised 11.9% of the sample, and the slope of the trajectory was cubic. The IQ = DLS group comprised 67.6% of the sample, and the slope of the trajectory was linear. Finally, the IQ > DLS group comprised 20.5% of the sample; the slope of the IQ > DLS group was quadratic.

### Aim 2: Characteristics associated with IQ‐DLS discrepancy trajectory groups

After corrections for multiple comparisons, there were no significant differences in demographic features (e.g., diagnostic status, race, and sex), ADOS CSS, or VIQ scores at age 9 by trajectory groups (Table 4). There were also no significant differences in adult internalizing or externalizing (Table S6). Given moderate classification uncertainty (Table S2), the group‐level comparisons described below should be interpreted with caution.

**Table 4:** Descriptive characteristics by DLS subdomain trajectory groups

	DLS domain		Personal subdomain			Domestic subdomain			Community subdomain		
	IQ < DLS	IQ > DLS	IQ < DLS	IQ = DLS	IQ > DLS	IQ < DLS	IQ = DLS	IQ > DLS	IQ < DLS	IQ = DLS	IQ > DLS
	*n* = 24	*n* = 103	*n* = 16	*n* = 95	*n* = 16	*n* = 17	*n* = 94	*n* = 18	*n* = 10	*n* = 95	*n* = 24
Demographic characteristics											
Sex (% female)	16.7	25.2	37.5	23.2	12.5	11.8	26.1	22.2	50	22.6	16.7
Diagnostic status (% DD^1^)	12.5	11.7	12.5	14.4	6.7	11.8	12	11.1	30	10.8	8.3
Race											
% White	75	61.2	50	63.2	81.3	64.7	59.6	77.8	80	58.9	70.8
% African American/Black	25	35.9	50	33.7	18.7	35.3	35.1	22.2	20	35.8	29.2
% Asian/Pacific Islander	0	1.9	0	0	0	0	0	0	0	2.1	0
% American Indian	0	0	0	2.1	0	0	2.1	0	0	1.1	0
% Multiracial	0	1	0	1	0	0	1.1	0	0	0	0
Urbanicity (% urban)	45.8	50	50	45.7	68.7	47	49.4	50	30	51	50
Caregiver education (% college education)	50	46.6	50	56.8	31.3	58.8	55.4	33.3	20	55.9	54.1
Language level (% Nonspeaking or minimally speaking)	66.7	83.1	75.0	81.4	75.0	88.9	82.1	58.8	66.7^a^	86.1^b^	60.0^a^
Phenotypic characteristics at age 9											
Profound categorization (% profound autism)	81.8	88.5	80.0	90.0	78.6	78.5	90.9	77.8	70.0	91.0	80.9
Verbal IQ	26.8 (16.7)	29.9 (17.7)	39.1 (14.8)	27.7 (17.3)	28.8 (18.5)	31.5 (14.2)^ab^	26.3 (17.6)^a^	38.9 (15.9)^b^	31.1 (16.7)	29.0 (18.0)	29.3 (16.9)
Nonverbal IQ	**38.8 (19.2)**	**62.4 (22.4)**	**49.4 (18.0)** ^**a**^	**37.3 (18.4)** ^**a**^	**71.6 (20.5)** ^**b**^	**41.6 (20.0)** ^**a**^	**40.0 (19.9)** ^**a**^	**65.8 (23.6)** ^**b**^	**52.8 (15.7)** ^**ab**^	**37.8 (19.2)** ^**b**^	**60.5 (24.3)** ^**a**^
Vineland ABC^2^	37.11 (12.0)	34.0 (11.5)	**42.5 (13.7)** ^**a**^	**32.0 (9.9)** ^**b**^	**38.4 (12.5)** ^**ab**^	37.5 (14.9)	33.3 (10.5)	37.5 (12.7)	**48.1 (12.9)** ^**a**^	**32.4 (9.6)** ^**b**^	**35.2 (12.5)** ^**b**^
ADOS CSS^3^	7.1 (2.7)	7.7 (1.8)	7.4 (2.6)	7.7 (1.7)	7.6 (2.7)	7.0 (2.7)	7.8 (1.8)	7.2 (2.5)	7.1 (3.1)	7.7 (1.8)	7.5 (2.4)
ADOS CSS—SA^4^	7.3 (2.5)	7.6 (1.8)	7.4 (2.6)	7.5 (1.7)	7.7 (2.3)	7.3 (2.3)	7.6 (1.8)	7.2 (2.4)	6.9 (3.0)	7.6 (1.8)	7.6 (2.1)
ADOS CSS—RRB^5^	7.2 (2.7)	7.9 (1.6)	7.4 (1.7)	7.9 (1.6)	7.5 (3.0)	6.6 (2.9)^a^	8.0 (1.6)^b^	7.5 (1.5)^ab^	7.6 (2.0)	7.9 (1.6)	7.2 (2.6)

Text in bold indicates significant group differences after corrections for multiple comparisons. Underlined text indicates significant group differences at *p* ≤ .05 (i.e., prior to corrections for multiple comparisons). At age 9, ratio IQ scores were calculated for 24.4% of participants (nonverbal IQ) and 27.5% of participants (verbal IQ) who completed IQ tests outside of age norms. Standardized IQ scores from age 9 are reported for all other participants, and nonverbal mental age was used to calculate all discrepancy trajectories. DLS, daily living skills; IQ, intelligent quotient. ^a,b^For each characteristic, mean values without common superscripts are significantly different after corrections for multiple comparisons.

^1^ Nonspectrum developmental disability.

^2^ Adaptive behavior composite standard scores.

^3^ Autism Diagnostic Observation Schedule, calibrated severity score.

^4^ Social affect.

^5^ Restricted, repetitive behaviors.

For the Personal and Domestic subdomains, participants in the IQ > DLS trajectory groups had significantly higher NVIQ at age 9 (both *p* < .001). NV IQ scores did not significantly differ between the IQ < DLS and IQ = DLS groups. For the Community subdomain, the IQ>DLS group had significantly higher nonverbal IQ scores than the IQ = DLS group; the IQ>DLS group did not significantly differ from either of the other two trajectory groups. Similarly, the type of cognitive test administered significantly varied across trajectory groups (Table 2). For the DLS Domain, participants in the IQ>DLS trajectory group were more likely to have completed a cognitive assessment higher on the IQ testing hierarchy than the MSEL (Mullen, 1995) than participants in the IQ < DLS trajectory group (*p* < .001). For the Personal and Community subdomains, participants in the IQ < DLS and IQ > DLS trajectory groups were more likely to have completed a cognitive assessment higher on the IQ testing hierarchy than the MSEL than participants in the IQ = DLS trajectory group (all *p* < .001).

Finally, there were significant differences in VABS ABC standard scores at age 9 by Personal and Community subdomain trajectory groups. For the Personal subdomain, participants in the IQ < DLS group had significantly higher ABC scores than participants in the IQ = DLS group (*p* < .001). Vineland ABC standard scores for participants in the IQ > DLS Personal trajectory group did not significantly differ from either of the other two trajectory groups. For the Community subdomain, participants in the IQ < DLS trajectory group had significantly higher Vineland ABC standard scores than participants in the IQ = DLS and IQ>DLS trajectory groups (all *p* < .001).

## Discussion

In all three DLS subdomains, a trajectory group with DLS that was stronger than IQ (as evidenced by large negative IQ‐DLS discrepancy scores; IQ < DLS) emerged, as well as a group with IQ that was stronger than DLS (as evidenced by large positive IQ‐DLS discrepancy scores; IQ>DLS). This three‐group model did not emerge when discrepancy scores were analyzed for the DLS domain as a whole (Figure 1). This underscores the importance of considering DLS subdomains separately when studying the discrepancy between cognitive ability and life skills in autism; domain‐level adaptive composites may obscure meaningful variability in real‐world functioning. From a clinical perspective, this highlights the need to assess and target life skills by domain—such as personal care, domestic routines, or community participation—rather than relying solely on overall adaptive scores.

For each DLS subdomain, approximately 80% of the sample was in either the IQ = DLS or the IQ < DLS trajectory group. In other words, most participants in this study had DLS commensurate with, or stronger than, their cognitive abilities across time. This contrasts with the existing literature, which suggests autistic individuals often have DLS that are weaker than their cognitive abilities (Braverman et al., 2024; Duncan & Bishop, 2015). Such research is often focused on autistic individuals with average or higher IQ and is based on cognitive and adaptive skills measured at a single time point. Our results underscore the heterogeneity of autism across both cognitive abilities and development.

Across all DLS subdomains, the IQ‐DLS discrepancy scores of the three trajectory groups were quite similar in early childhood (Table 2). By mid‐childhood, IQ‐DLS discrepancies in this sample were apparent and persisted into adulthood. Notably, the discrepancy scores of the IQ>DLS trajectory groups became larger with increasing age (i.e., NV abilities growth outpaced DLS growth more as participants became older). In comparison, discrepancy scores in the IQ<DLS trajectory groups became more negative with increasing age (i.e., DLS growth outpaced NV abilities growth more as participants got older).

It remains unclear why IQ‐DLS discrepancies exist in autism. Notably, one study of autistic adolescents with average or better IQ found that only 10% of the apparent “gap” in DLS and cognitive skills was explained by age, maternal education, sex, and autism symptomology (Duncan & Bishop, 2015). This suggests that demographic and some individual‐level characteristics are not strongly related to DLS. Rather, that the majority of participants fell in the IQ = DLS and IQ < DLS trajectory groups seems to suggest the importance of life skills interventions.

Emerging evidence suggests that opportunity may be one factor to consider in DLS development. In autistic 16–35‐year‐olds with and without ID, Teh et al. (2024) found that, after cognitive ability, lack of opportunity was the most common reason VABS‐3 DLS items were rated 0, indicating the individual “Never or Seldom” completed that skill independently. The IQ < DLS trajectory groups may reflect increasing mastery of DLS when given sufficient chances to learn and practice. On the other hand, perhaps participants in the IQ > DLS trajectory groups did not have sufficient opportunities to learn DLS that were at least commensurate with their cognitive abilities. Providing sufficient practice to learn new DLS and master emerging skills, ideally across a range of contexts (e.g., clinic, home, school), could support DLS growth that is commensurate with, or even exceeds, growth in cognitive abilities in autistic individuals with co‐occurring ID.

The later emergence and increasing nature of discrepancies could be a measurement artifact. However, such findings may also suggest that, beyond opportunity to learn and practice, supporting adaptive skill development in autistic individuals with ID may require *domain‐specific* and *developmentally timed* approaches. For example, interventions targeting community and domestic skills may be most impactful when introduced during late adolescence and early adulthood, as these are periods when environmental expectations rise, but learning opportunities may decline. These results also suggest that clinical services and educational programs should track adaptive gains within subdomains rather than relying solely on global indices to better tailor supports and monitor progress over time.

There was some evidence that inclusion in the IQ = DLS trajectory group was associated with higher support needs. In all three subdomains, participants in the IQ > DLS group had significantly higher NVIQ (but not VIQ) scores than participants in the IQ = DLS group. For the Personal and Community subdomains, inclusion in the IQ = DLS trajectory group was associated with a higher likelihood of only completing the MSEL, used to estimate IQ in those with the greatest level of ID (Table 2; Anderson et al., 2014). Individuals with very low values for NV estimates and DLS AEs would have discrepancy scores close to zero and thus would be more likely to be included in the IQ = DLS trajectory group. However, there was variability in the IQ‐DLS discrepancy scores of participants in the IQ = DLS groups across subdomains (Table 3, Tables S3–S5), indicating that not all participants in the IQ = DLS groups had profound ID.

This sample included a subset of participants who were not autistic but were diagnosed with nonspectrum developmental conditions in early childhood. Notably, diagnostic status did not significantly differ across any of the identified trajectory groups. This null finding parallels prior analyses of this longitudinal cohort, which have found evidence for many similarities in the characteristics and experiences of autistic individuals and those with other developmental conditions (Clarke & Lord, 2025; Lord et al., 2020). Together, these results suggest that early developmental pathways related to cognitive and adaptive functioning may be shaped by shared developmental characteristics and contextual factors rather than categorical diagnostic distinctions alone.

Associations between internalizing and externalizing symptoms and DLS trajectories did not survive correction for multiple comparisons. Notably, however, the observed patterns (see Appendix S3) align with prior work in autistic individuals with average or higher cognitive abilities, indicating that relatively strong DLS are associated with low internalizing symptoms (Donoso et al., 2024; Gardiner, 2018). The current null findings may suggest that the interplay between adaptive and mental health outcomes differs for autistic individuals with co‐occurring ID compared to autistic individuals with average or better IQ. However, only a small subsample of participants had data on mental health measures (Table S6). Future research in larger samples exploring associations between mental health and DLS discrepancies in autistic individuals with co‐occurring ID is warranted.

### Limitations

In prior studies of autistic individuals with average or higher IQ, an IQ‐DLS discrepancy was defined as a difference of at least one standard deviation (i.e., 15 points) in standard scores (Duncan & Bishop, 2015). There are no published standard deviations or confidence intervals for VABS or cognitive test AEs, so it is less straightforward to define an IQ‐DLS discrepancy in the current sample. The data‐driven analytic design used here somewhat accounts for this; rather than defining an IQ‐DLS discrepancy between NV abilities and DLS AE a priori, group‐based trajectory analyses identified latent profiles of IQ‐DLS discrepancy scores within the sample. However, the use of this approach limits the ability to compare the results of the present study to the existing literature on IQ‐DLS discrepancies in autistic individuals of average or better IQ. Further, though this study focused on discrepancy direction rather than magnitude, future work should examine the functional impact of smaller (e.g., 25‐month) versus larger (e.g., 50‐month) discrepancies.

The current study used NVMA to measure NV abilities. Ratio IQ scores decrease with increasing chronological age and thus may provide an underestimate of cognitive abilities, particularly in adulthood (Bishop et al., 2015). Though standardized IQ scores are always preferable, no cognitive tests are adequately standardized for use across the life course with individuals with severe to profound ID. Given that the measures predominantly used to calculate NV abilities (MSEL and DAS‐II; Table 2) are not normed in adults, it is difficult to determine whether these scores accurately represent the cognitive abilities of the current sample in adulthood.

Measuring DLS in autistic adults also presents challenges. Importantly, AE estimates are not an equal‐interval measure and may function more like an ordinal scale than a continuous one. For example, the difference in DLS abilities between two individuals with AE estimates of 2 and 3 years is quite different from the difference in DLS abilities between two individuals with estimates of 9 and 10 years, even though the interval (12 months) is the same. As a result, the discrepancy scores presented here may not be psychometrically consistent or comparable across ages; this is an unavoidable consequence of using AE estimates to calculate discrepancy scores.

Although widely used, the VABS may not be an optimal measure for assessing DLS in adults. Only 14.4% of the standardization sample for the VABS‐II was over age 22 (Sparrow et al., 2005), resulting in VABS AEs becoming sparser with increasing age and a maximum VABS age equivalent of 264 months (22 years). For example, a 1‐point difference in raw scores in the Personal subdomain on the VABS‐II can result in a 3+ year difference in AEs, which may overemphasize discrepancies at the higher skill levels and limit the utility of the VABS for measuring DLS in autistic adults. However, it is unlikely that ceiling effects influenced these results, as the maximum DLS AE in the current sample was 210 months (17.5 years) and the maximum NV estimate was 199.26 months (16.61 years).

This sample comprises a unique group of adults with developmental conditions, ascertained in the early 1990s. These participants may be different from both younger individuals and same‐aged individuals diagnosed later in development (Lord et al., 2020). There are relatively few female participants and participants of color, which constrained the ability to test for differences by sex and race. While attrition in this longitudinal cohort is comparable to other population‐based (Gustavson, von Soest, Karevold, & Røysamb, 2012) and autism‐specific (Taylor & Mailick, 2014) longitudinal samples, missing data at later time points may have impacted these findings. Notably, trajectory estimations are often sensitive to data from later time points (Mésidor, Rousseau, O'Loughlin, & Sylvestre, 2022), and the two adult data points in this study, ages 18 and 25, had higher proportions of missing data (41.1% and 67.7% respectively, see Table 3). The present results should be interpreted with this in mind. Similarly, because entropy values reflected moderate classification certainty, the group comparisons reported here may be affected by some degree of classification error and should be interpreted with appropriate caution.

## Conclusion

To our knowledge, this is the first longitudinal study of IQ‐DLS discrepancies in autistic adults with ID and the first to examine discrepancies in subdomains (i.e., Personal, Domestic, Community). By identifying both shared and domain‐specific developmental patterns, this study suggests that DLS does not develop uniformly across domains. The majority (80%) of participants had either DLS commensurate (IQ = DLS) or notably stronger (IQ < DLS) than their cognitive abilities, highlighting the importance of ongoing opportunities and supports to learn and practice skills throughout adolescence and adulthood. These results underscore the importance of including autistic individuals with ID in research, as patterns observed in samples with average or higher IQ may not be generalizable. Future studies should also explore IQ‐DLS trajectories in individuals with average or higher IQ and how opportunities for learning, as well as other factors, impact DLS attainment across development.

## Ethical considerations

Parents and participants over 18 years who were their own legal guardians gave written consent as required by the institutional review board prior to visits. This study (IRB‐19‐0079) was approved by the University of California, Los Angeles Medical IRB 3 on March 7, 2025.

**Key points**

**What's known?**

- Daily living skills (DLS) are essential for independence and quality of life, but are often reported to lag behind cognitive abilities in autistic individuals without intellectual disability.

**What's new?**

- This is the first study to examine longitudinal trajectories of IQ‐DLS discrepancies in autistic individuals with co‐occurring intellectual disability.
- Most participants had DLS similar to or stronger than cognitive abilities across development, challenging assumptions that DLS data are uniformly impaired in autism.

**What's relevant?**

- In clinical contexts, understanding how cognitive abilities and DLS develop in relation to one another is important to supporting autistic individuals, as is providing sufficient opportunities to learn and master DLS.

## Supporting information


**Appendix S1.** Descriptive characteristics of the current sample (*n* = 127) compared to the total longitudinal cohort (*n* = 253).
**Appendix S2.** IQ‐DLS discrepancy trajectories in autistic participants only (*n* = 112).
**Appendix S3.** Internalizing and externalizing measures.
**Figure S1.** Trajectories of NV abilities – DLS AE domain and subdomain discrepancy scores from ages 2 to 25 in autistic participants only.
**Table S1.** IQ‐DLS discrepancy trajectories model selection in autistic participants only (*n* = 112).
**Table S2.** IQ‐DLS discrepancy trajectories model selection (*n* = 127).
**Table S3.** NVMA and DLS AE by personal subdomain trajectory groups.
**Table S4.** NVMA and DLS AE by domestic subdomain trajectory groups.
**Table S5.** NVMA and DLS AE by community subdomain trajectory groups.
**Table S6.** Adult Mental Health Characteristics of DLS subdomain trajectory groups.

## Data Availability

The data that support the findings of this study are available upon request from qualified researchers in the National Institute of Mental Health Data Archive (NDA) at https://nda.nih.gov/.

## Endnote

1 The cohort includes both autistic participants and a subset of participants identified between ages 2 and 3 as having nonspectrum developmental delays who were not diagnosed with autism at any timepoint. For brevity, the term autistic individuals is used throughout to refer to the full cohort.
