# Supplementary material for: Relative strengths in daily living skills among autistic individuals and individuals with related developmental conditions who have co‐occurring intellectual disability
Source: J Child Psychol Psychiatry. 2026 Jan 28;67(7):1147–58. doi: 10.1111/jcpp.70124 (PMC13265627; doi:10.1111/jcpp.70124)
Supplement: Supplementary file 1 — Appendix S1. Descriptive characteristics of the current sample (n = 127) compared to the total longitudinal cohort (n = 253). Appendix S2. IQ‐DLS discrepancy trajectories in autistic participants only (n = 112). Appendix S3. Internalizing and externalizing measures. Figure S1. Trajectories of NV abilities – DLS AE domain and subdomain discrepancy scores from ages 2 to 25 in autistic participants only. Table S1. IQ‐DLS discrepancy trajectories model selection in autistic participants only (n = 112). Table S2. IQ‐DLS discrepancy trajectories model selection (n = 127). Table S3. NVMA and DLS AE by personal subdomain trajectory groups. Table S4. NVMA and DLS AE by domestic subdomain trajectory groups. Table S5. NVMA and DLS AE by community subdomain trajectory groups. Table S6. Adult Mental Health Characteristics of DLS subdomain trajectory groups. [file JCPP-67-1147-s001.docx]

**Relative Strengths in Daily Living Skills Among Autistic Individuals and Individuals with Related Developmental Conditions who have Co-Occurring Intellectual Disability**

**Supporting Information**

**Appendix S1. Descriptive Characteristics of the Current Sample (n = 127) Compared to the Total Longitudinal Cohort (n = 253)**

Participants in the current study did not significantly differ from the total longitudinal cohort by sex X^2^(1, 253) = 2.25, p = .13, ethnicity X^2^(1, 250) = 2.74, p = .09, race  X^2^(1, 253) = 3.48, p = .06, urbanicity X^2^(1, 253) = 2.84, p = .09, or maternal education X^2^(1, 253) = .35, p = .550. Compared to the total longitudinal cohort, participants in the current analyses had significantly lower verbal IQ scores t(178) = 20.61, p < .001, and significantly higher Autism Diagnostic Observation Schedule (ADOS) calibrated severity scores (CSS) t(194) = 9.27, p < .001. They were also significantly more likely to have received an autism diagnosis X^2^(1, 253) = 12.75, p < .001, and were significantly more likely to have been enrolled in the study from the North Carolina or Chicago sites X^2^(2, 253) = 30.29, p < .001.

**Appendix S2. IQ-DLS Discrepancy Trajectories in Autistic Participants Only (n = 112)**

***Personal Subdomain***

The IQ < DLS trajectory group comprised 23.2% of the sample, and the slope of the IQ < DLS trajectory was quadratic. The IQ < DLS group was characterized by IQ-DLS discrepancy scores that declined with age. The IQ = DLS group comprised 60.8% of the sample, and the slope of the IQ=DLS group was linear. This group was characterized by IQ-DLS discrepancy scores close to zero that changed little with age. Finally, the IQ > DLS group comprised 15.9% of the sample; the slope of the IQ > DLS group was quadratic. This group was characterized by IQ-DLS discrepancy scores that increased from ages 2 to 18, then declined from ages 18 to 25. However, IQ-DLS discrepancy scores remained positive from 18 to 25 (Figure S1).

***Domestic Subdomain***

In contrast to the Domestic subdomain group-based trajectory analysis that included both autistic participants and participants with a non-spectrum developmental condition, in which a three-group model emerged as the best fit, a two-group model best fit the data for autistic participants (Table S1). There was no IQ > DLS trajectory group in this model, but IQ = DLS and IQ < DLS groups only. The IQ < DLS trajectory group comprised 30.2% of the sample, and the slope of the trajectory was quadratic. IQ-DLS discrepancy scores declined from ages 2-25. The IQ = DLS group comprised 69.8% of the sample, and the slope of the IQ=DLS group was also quadratic. This group was characterized by IQ-DLS discrepancy scores close to zero, however, scores did decrease slightly from ages 18 to 25 (Figure S2).

***Community Subdomain***

The IQ < DLS trajectory group comprised 7.1% of the sample and the slope of the trajectory was quadratic. IQ-DLS discrepancy scores in the IQ < DLS group declined from ages 2-18, then continued to decline at a slower rate from 18-25. The IQ = DLS group comprised 69.1% of the sample, and the slope of the trajectory was linear. This group was characterized by IQ-DLS discrepancy scores close to zero at all timepoints. Finally, the IQ > DLS group comprised 23.8% of the sample; the slope of the IQ > DLS group was linear This group was characterized by IQ-DLS discrepancy scores that increased from ages 2 to 25 (Figure S3).

Table S1. IQ-DLS Discrepancy Trajectories Model Selection in Autistic Participants Only (n = 112)

|  |  | **DLS Domain** | | | | **Personal Subdomain** | | | |  |
| --- | --- | --- | --- | --- | --- | --- | --- | --- | --- | --- |
|  | **Model** | BIC | AIC | Smallest Group % | Entropy | BIC | AIC | Smallest Group % | Entropy | |
|  | 1 Class Model | -1873.75 | -1869.67 | — | — | -1914.17 | -2160.77 | — | — | |
|  | 2 Class Model | **-2131.61** | **-1854.26** | **25.48** | **0.74** | -1854.26 | -2107.47 | 13.75 | 0.64 | |
|  | 3 Class Model | -1785.30 | -1773.06 | 6.62 | 0.72 | **-1814.99** | **-2035.75** | **14.13** | **0.76** | |
|  | 4 Class Model | -1775.46 | -1759.15 | 5.46 | 0.68 | -1836.47 | -2055.62 | 3.29 | 0.69 | |
|  |  | **Domestic Subdomain** | | | | **Community Subdomain** | | | | |
|  | **Model** | BIC | AIC | Smallest Group % | Entropy | BIC | AIC | Smallest Group % | Entropy | |
|  | 1 Class Model | -2025.60 | -2019.45 | — | — | -1912.07 | -1905.93 | — | — | |
|  | 2 Class Model | **-1969.49** | **-1953.09** | **30.24** | **0.62** | -1885.16 | -1872.88 | 10.48 | 0.67 | |
|  | 3 Class Model | -1988.56 | -1970.11 | 0.00 | 0.63 | **-1860.09** | **-1839.62** | **7.05** | **0.78** | |
|  | 4 Class Model | — | — | — | — | -1856.37 | -1831.81 | 3.96 | 0.65 | |

Note: Final model selected in bold.

Figure S1. Trajectories of NV Abilities – DLS AE Domain and Subdomain Discrepancy Scores from Ages 2-25 in Autistic Participants Only


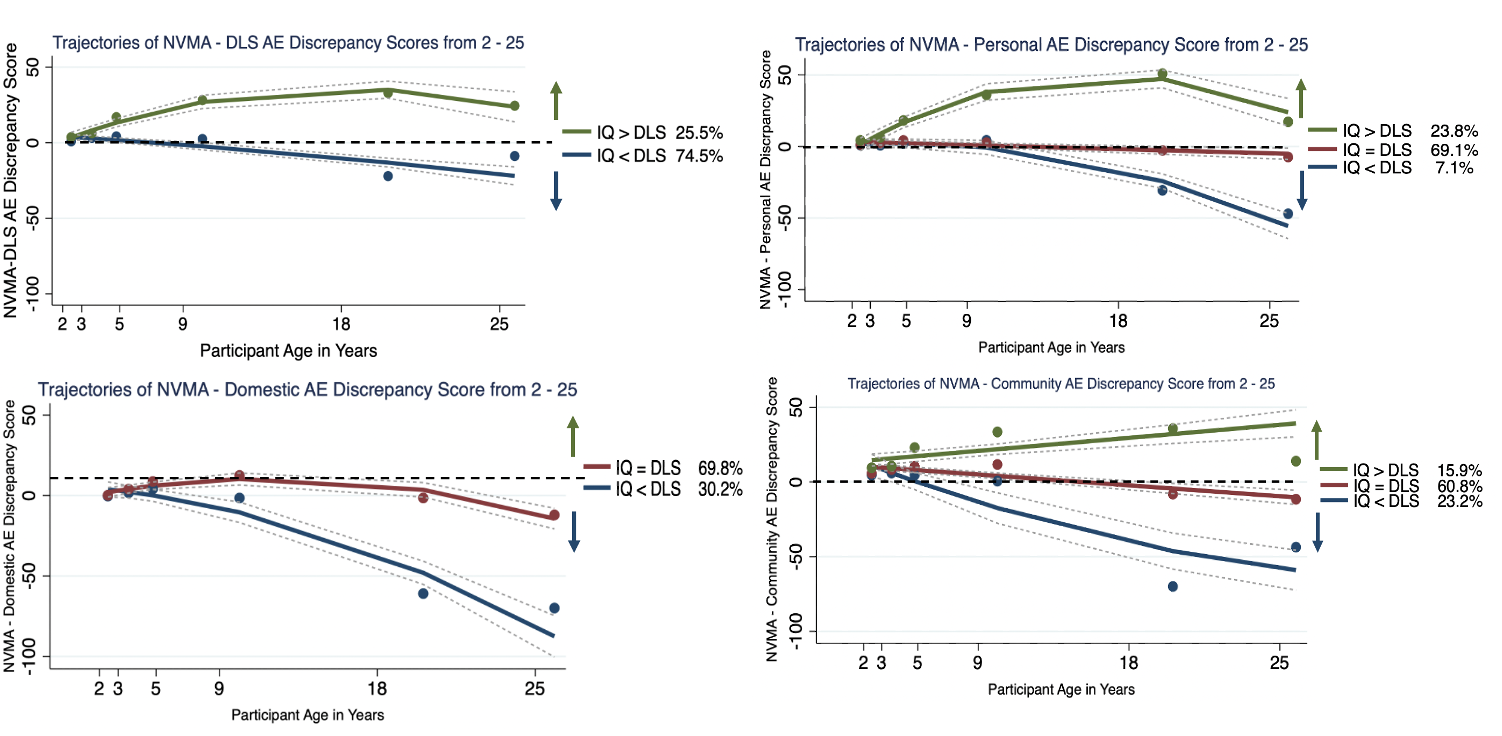


|  |  | **DLS Domain** | | | | **Personal Subdomain** | | | | |  |
| --- | --- | --- | --- | --- | --- | --- | --- | --- | --- | --- | --- |
|  | **Model** | BIC | AIC | Smallest Group % | Entropy | BIC | AIC | Smallest Group % | Entropy |  | |
|  | 1 Class Model | -2193.60 | -2191.50 | — | — | -2167.10 | -2160.77 | — | — |  | |
|  | 2 Class Model | **-2131.61** | **-2127.39** | **22.51** | **0.79** | -2111.60 | -2107.47 | 13.75 | 0.81 |  | |
|  | 3 Class Model | -2110.78 | -2091.66 | 5.48 | 0.70 | **-2058.96** | **-2035.75** | **14.13** | **0.84** |  | |
|  | 4 Class Model | — | — | — | — | -2080.95 | -2055.62 | 3.29 | 0.77 |  | |
|  |  | **Domestic Subdomain** | | | | **Community Subdomain** | | | |  | |
|  | **Model** | BIC | AIC | Smallest Group % | Entropy | BIC | AIC | Smallest Group % | Entropy |  | |
|  | 1 Class Model | -2299.38 | -2293.05 | — | — | -2181.94 | -2175.62 | — | — |  | |
|  | 2 Class Model | -2245.20 | -2232.54 | 21.41 | 0.67 | -2146.44 | -2133.80 | 12.53 | 0.78 |  | |
|  | 3 Class Model | **-2196.53** | **-2171.21** | **16.10** | **0.67** | **-2073.75** | **-2048.47** | **11.92** | **0.73** |  | |
|  | 4 Class Model | -2216.43 | -2191.10 | 10.23 | 0.58 | -2105.37 | -2080.08 | 8.72 | 0.67 |  | |
|  | 5 Class Model | -2224.70 | -2193.05 | 0.00 | 0.66 | -2114.69 | -2083.08 | 0.00 | 0.71 |  | |

Table S2. IQ-DLS Discrepancy Trajectories Model Selection (n = 127)

Note: Final model selected in bold.

Table S3. NVMA and DLS AE by Personal Subdomain Trajectory Groups

|  |  | **Data Collection Timepoint** | | | | | |
| --- | --- | --- | --- | --- | --- | --- | --- |
| **n** | | **2** | **3** | **5** | **9** | **18** | **25** |
|  |  | 115 | 102 | 77 | 98 | 73 | 40 |
|  |  | **m (SD)**  **[range]** | | | | | |
| **IQ>DLS** | **NV Abilities** | 21.20 (5.49)  [13.20-32.20] | 27.27 (6.48)  [14.40-37.96] | 47.09 (14.05)  [26.79-66.00] | 80.12 (25.70)  [32.0-117.04] | 135.90 (37.55)  [79.0-199.26] | 90.50 (15.0)  [76.0-110.0] |
|  | **Personal AE** | 17.21 (7.66)  [10.0-33.0] | 20.50 (7.24)  [13.0-39.0] | 28.50 (10.20)  [19.0-45.0] | 46.46 (17.40)  [26.0-87.0] | 84.18 (40.36)  [19.0-138.0] | 72.0 (20.70)  [49.0-90.0] |
| **IQ=DLS** | **NV Abilities** | 16.59 (5.79)  [3.90-30.45] | 21.73 (6.84)  [7.14-37.50] | 27.55 (11.26)  [6.90-59.0] | 41.16 (19.68)  [2.34-89.25] | 56.02 (34.96)  [6.0-155.44] | 51.81 (40.15)  [6.0-186.60] |
|  | **Personal AE** | 14.91 (4.49)  [4.0-27.0] | 19.34 (5.59)  [8.0-35.0] | 23.59 (6.97)  [9.0-43.0] | 37.59 (16.32)  [11.0-105.0] | 59.26 (38.20)  [3.0-180.0] | 58.96 (37.21)  [17.0-180.0] |
| **IQ<DLS** | **NV Abilities** | 15.81 (4.87)  [6.96-24.65] | 22.96 (5.77)  [16.50-32.90] | 31.09 (9.40)  [19.80-51.46] | 56.31 (21.87)  [19.0-98.40] | 61.56 (30.37)  [4.0-109.0] | 61.63 (25.49)  [23.0-91.0] |
|  | **Personal AE** | 14.50 (5.27)  [3.0-26.0] | 22.75 (3.19)  [19.0-29.0] | 29.73 (6.0)  [18.0-38.0] | 51.14 (14.09)  [28.0-76.0] | 99.38 (39.47)  [27.0-180.0] | 112.88 (31.83)  [60.0-138.0] |

Note. The posterior probabilities for the Personal subdomain trajectory groups were as follows: IQ>DLS = 0.90, IQ=DLS = 0.77, IQ<DLS = .93.

Table S4. NVMA and DLS AE by Domestic Subdomain Trajectory Groups

|  |  | **Data Collection Timepoint** | | | | | |
| --- | --- | --- | --- | --- | --- | --- | --- |
| **n** | | **2** | **3** | **5** | **9** | **18** | **25** |
|  |  | 115 | 102 | 77 | 98 | 73 | 40 |
|  |  | **m (SD)**  **[range]** | | | | | |
| **IQ>DLS** | **NV Abilities** | 20.32 (6.39)  [9.80-32.20] | 25.14 (8.04)  [12.80-37.96] | 38.80 (10.33)  [22.44-52.0] | 75.61 (25.70)  [40.0-117.04] | 134.12 (43.34)  [53.0-199.26] | 118.15(47.05)  [82.0-186.60] |
|  | **Domestic AE** | 17.60 (3.50)  [16.0-27.0] | 19.71 (6.30)  [16.0-36.0] | 24.0 (9.24)  [16.0-42.0] | 43.23 (20.64)  [16.0-91.0] | 94.80 (33.30)  [30.0-144.0] | 84.75 (32.68)  [47.0-126.0] |
| **IQ=DLS** | **NV Abilities** | 16.72 (5.54)  [5.32-28.35] | 22.06 (6.39)  [7.70-34.20] | 28.95 (12.93)  [6.90-66.0] | 44.66 (22.20)  [2.34-104.49] | 59.99 (35.52)  [4.0-165.0] | 54.71 (30.70)  [6.0-118.0] |
|  | **Domestic AE** | 16.68 (2.03)  [16.0-24.0] | 18.69 (3.67)  [16.0-30.0] | 22.98 (8.57)  [16.0-61.0] | 39.64 (18.99)  [16.0-91.0] | 78.15 (39.83)  [11.0-168.0] | 74.14 (44.42)  [11.0-183.0] |
| **IQ<DLS** | **NV Abilities** | 16.03 (6.10)  [3.90-26.24] | 23.06 (8.0)  [7.14-37.55] | 27.72 (10.26)  [13.20-51.46] | 45.81 (22.97)  [18.0-98.40] | 56.23 (33.75)  [18.0-98.40] | 37.63 (23.84)  [18.0-91.0] |
|  | **Domestic AE** | 17.56 (5.07)  [16.0-36.0] | 22.86 (11.97)  [16.0-61.0] | 24.90 (12.06)  [16.0-57.0] | 49.18 (19.68)  [21.0-86.0] | 134.0 (57.15)  [77.0-210.0] | 110.0 (29.40)  [59.0-144.0] |

Note. The posterior probabilities for the Domestic subdomain trajectory groups were as follows: IQ>DLS = 0.90, IQ=DLS = 0.82, IQ<DLS = .84.

Table S5. NVMA and DLS AE by Community Subdomain Trajectory Groups

|  |  | **Data Collection Timepoint** | | | | | |
| --- | --- | --- | --- | --- | --- | --- | --- |
| **n** | | **2** | **3** | **5** | **9** | **18** | **25** |
|  |  | 115 | 102 | 77 | 98 | 73 | 40 |
|  |  | **m (SD)**  **[range]** | | | | | |
| **IQ>DLS** | **NV Abilities** | 20.72 (5.60)  [12.47-32.20] | 26.36 (7.08)  [14.40-37.96] | 44.34 (13.36)  [22.44-66.0] | 68.11 (28.66)  [22.31-117.0] | 125.80 (48.06)  [24.0-199.26] | 106.43 (40.84)  [78.0-186.6] |
|  | **Community AE** | 10.74 (4.93)  [7.0-17.0] | 15.05 (6.97)  [7.0-32.0] | 19.08 (11.30)  [7.0-37.0] | 32.26 (20.64)  [7.0-71.0] | 88.07 (41.14)  [7.0-168.0] | 79.0 (24.88)  [38.0-114.0 |
| **IQ=DLS** | **NV Abilities** | 16.24 (5.67)  [3.90-28.35] | 21.63 (6.60)  [7.14-37.50] | 26.64 (10.54)  [6.90-59.0] | 41.65 (20.75)  [2.34-96.0] | 53.60 (32.03)  [4.0-136.0] | 47.50 (30.87)  [6.0-118.0] |
|  | **Community AE** | 10.14 (5.75)  [7.0-26.0] | 13.61 (5.97)  [7.0-29.0] | 16.66 (7.51)  [7.0-48.0] | 31.22 (19.63)  [7.0-81.0] | 60.14 (32.54)  [7.0-141.0] | 62.60 (30.78)  [11.0-118.0] |
| **IQ<DLS** | **NV Abilities** | 16.77 (5.14)  [11.28-28.13] | 22.30 (6.44)  [16.45-32.90] | 34.21 (10.19)  [24.38-51.46] | 61.11 (18.95)  [42.0-98.40] | 66.90 (22.90)  [41.0-106.0] | 56.17 (24.14)  [31.0-91.0] |
|  | **Community AE** | 10.80 (5.35)  [7.0-17.0] | 16.43 (5.38)  [7.0-21.0] | 26.50 (9.5)  [17.0-40.0] | 56.44 (22.10)  [37.0-110.0] | 134.70 (51.78)  [82.0-195.0] | 97.50 (15.23)  [82.0-118.0] |

Note. The posterior probabilities for the Community subdomain trajectory groups were as follows: IQ>DLS = 0.83, IQ=DLS = 0.89, IQ<DLS = .93.

**Appendix S3. Internalizing & Externalizing Measures**

Caregivers were asked to complete questionnaires of participants’ co-occurring internalizing and externalizing symptoms in adulthood. Internalizing was measured using the Anxiety and Depression subscales of the Anxiety, Depression, and Mood Scale (ADAMS; (Esbensen et al., 2003) and the Internalizing subscale of the Adult Behavior Checklist (ABCL; Achenbach & Rescorla, 2003). Externalizing was measured using the Hyperactivity and Irritability subscales of the Aberrant Behavior Checklist (ABC; Aman et al., 1985) and the externalizing subscale of the ABCL. ABCL data were collected at approximately age 26 (m_age_=26.73, SD=3.91). ADAMS and ABC data were collected at approximately age 21 (m_age_=21.86, SD=6.15).

**Mental Health Characteristics by DLS Subdomain IQ-DLS Trajectory Groups**

After Benjamini-Hochberg corrections, there were no significant differences in participants’ mental health characteristics based on trajectory group membership. Exploratory analyses suggested small, non-significant trends by trajectory group; these are reported below for completeness.

***Personal Subdomain***

Scores on measures of internalizing and externalizing in adulthood did not significantly differ across Personal subdomain trajectory groups (all *p* > .05; Table S6).

***Domestic Subdomain***

There were no differences across Domestic trajectory groups on externalizing measures or ADAMS anxiety scores (all *p* > .05). However, prior to corrections for multiple comparisons, ABCL internalizing, *F*(2,51)=3.46, *p*=.039, ηp²=.12, and ADAMS Depression scores *F*(2,52)=4.54, *p*=.015, ηp²=.15, did significantly differ (Table S6). Post-hoc analyses revealed that participants in the IQ>DLS trajectory group had significantly higher ADAMS Depression scores than participants in IQ=DLS (*p* =.033) and IQ<DLS (*p* =.014) groups. Similar patterns of ABCL internalizing scores were observed (IQ>DLS higher than IQ=DLS (*p* =.058) and IQ<DLS groups (*p* =.050)).

***Community Subdomain***

There were no differences in externalizing measures or ADAMS anxiety scores across Community subdomain trajectory groups (all *p* > .05). However, prior to corrections for multiple comparisons, ABCL internalizing, *F*(2,51)=3.42, *p*=.040, ηp²=.12, and ADAMS Depression scores *F*(2,52)=3.37, *p*=.042, ηp²=.12, did significantly differ (Table S6). Post-hoc analyses revealed a similar pattern as observed for the Domestic subdomain groups (i.e., IQ>DLS trajectory group higher ADAMS Depression (*p* =.052) and ABCL internalizing (p=.035) scores than participants in IQ=DLS group, but no difference between IQ>DLS and IQ<DLS groups (*p* =.60, *p* =.45, respectively).

|  | **DLS Domain** | | **Personal Subdomain** | | | **Domestic Subdomain** | | | **Community Subdomain** | | |
| --- | --- | --- | --- | --- | --- | --- | --- | --- | --- | --- | --- |
|  | IQ<DLS  n = 19 | IQ>DLS  n = 87 | IQ<DLS  n = 16 | IQ=DLS  n = 95 | IQ>DLS  n = 16 | IQ<DLS  n = 17 | IQ=DLS  n = 94 | IQ>DLS  n = 18 | IQ<DLS  n = 10 | IQ=DLS  n = 95 | IQ>DLS  n = 24 |
| **ADAMS**^1^ **(n = 54)** | **m(SD)** | |  | **m(SD)** |  |  | **m(SD)** |  |  | **m(SD)** |  |
| Depression Subscale | 3.9(6.3) | 2.5(3.3) | 1.4(2.6) | 3.4(4.5) | 2.5(2.2) | 1.57(1.6)^a^ | 2.6(3.2)^a^ | 7.0(8.3)^b^ | 3.9(4.2) | 1.95(2.5) | 5.3(6.8) |
| Anxiety Subscale | 5.8(5.2) | 4.0(4.0) | 3.7(3.1) | 4.8(4.7) | 4.5(2.6) | 3.4(1.9) | 4.5(4.6) | 7.2(5.5) | 4.5(4.2) | 3.9(4.0) | 6.7(4.8) |
| **ABC**^2^ **(n = 53)** |  |  |  |  |  |  |  |  |  |  |  |
| Hyperactivity Subscale | 10.4(9.4) | 10.5(10.9) | 9.4(12.7) | 11.2(10.6) | 10.4(9.2) | 8.8(8.6) | 11.5(11.5) | 10.2(9.4) | 4.5(5.1) | 11.7(11.4) | 10.9(9.2) |
| Irritability Subscale | 8.8(6.8) | 8.3(8.9) | 6.4(7.2) | 9.2(9.1) | 7.6(5.3) | 5.7(5.5) | 9.2(9.2) | 9.0(7.3) | 2.7(2.7) | 9.2(9.1) | 9.3(7.0) |
| **ABCL**^3^ **(n = 94)** |  |  |  |  |  |  |  |  |  |  |  |
| Internalizing Subscale | 53.6(9.2) | 50.4(7.4) | 48.0(7.7) | 51.6(7.9) | 51.8(8.6) | 49.0(7.2) | 49.9(7.6) | 57.7(7.9) | 51.1(8.0)^ab^ | 49.1(7.3)^a^ | 56.2(8.3)^b^ |
| Externalizing Subscale | 55.5(9.0) | 51.9(7.1) | 52.9(8.5) | 53.8(7.1) | 49.5(6.8) | 52.4(4.3) | 52.6(7.8) | 56.7(9.3) | 52.8(4.5) | 52.4(7.5) | 55.9(9.3) |

Note. Underlined text indicates significant group differences at p < .05. These group differences did not survive corrections for multiple comparisons.

^a,b^For each trajectory group, mean values without common superscripts are significantly different (*p* < .05).

^1^Anxiety, Depression, and Mood Scale (ADAMS; (Esbensen et al., 2003)

^2^Aberrant Behavior Checklist (ABC Aman et al., 1985)

^3^Adult Behavior Checklist (ABCL; Achenbach & Rescorla, 2003)

Table S6. Adult Mental Health Characteristics of DLS Subdomain Trajectory Groups
